# Constructing Adult Zebrafish Einthoven’s Triangle to Define Electrical Heart Axes

**DOI:** 10.3389/fphys.2021.708938

**Published:** 2021-07-23

**Authors:** Yali Zhao, Connie Chen, Morgan Yun, Thomas Issa, Andrew Lin, Thao P. Nguyen

**Affiliations:** The Cardiovascular Research Laboratory, Division of Cardiology, Department of Medicine, David Geffen School of Medicine at University of California, Los Angeles, Los Angeles, CA, United States

**Keywords:** zebrafish, Einthoven’s triangle, electrical heart axis, main heart axis, electrocardiogram, bipolar dual-lead ECG, Cabrera system

## Abstract

Zebrafish is a popular high-throughput vertebrate model to study human cardiac electrophysiology, arrhythmias, and myopathies. One reason for this popularity is the purported striking similarities between zebrafish and human electrocardiograms (ECGs). However, zebrafish electrical heart axes were unknown. It is impossible to define heart axis based on single-lead ECG because determination of an electrical heart axis in the frontal plane requires the use of the hexaxial reference system (or Cabrera system) derived from Einthoven’s triangle. Construction of Einthoven’s triangle requires simultaneous ECG recording from at least two Einthoven bipolar leads. Therefore, we systematically constructed the first zebrafish Einthoven’s triangle by simultaneous bipolar dual-lead ECG recording to determine for the first time the three frontal electrical heart axes using the Cabrera system. Comparing zebrafish with human Einthoven’s triangle reveals that their normal frontal electrical axes were reflections of each other across 0° in the Cabrera system. The responsible mechanisms involve zebrafish vs. human cardiac activation propagating in the same direction along the heart horizontal axis but in opposite directions along the heart longitudinal axis. The same observations are true for zebrafish vs. human cardiac repolarization. This study marks a technical breakthrough in the first bipolar dual-lead ECG recording in live adult zebrafish to construct for the first time zebrafish Einthoven’s triangle. This first systematic analysis of the actual differences and similarities between normal adult zebrafish and human Einthoven’s triangles unmasked differences and similarities in the underlying cardiac axis mechanisms. Insights of the live adult zebrafish main heart axis and its three frontal electrical heart axes provide critical contextual framework to interpret the clinical relevance of the adult zebrafish heart as model for human cardiac electrophysiology.

## Introduction

**FIGURE 1:**
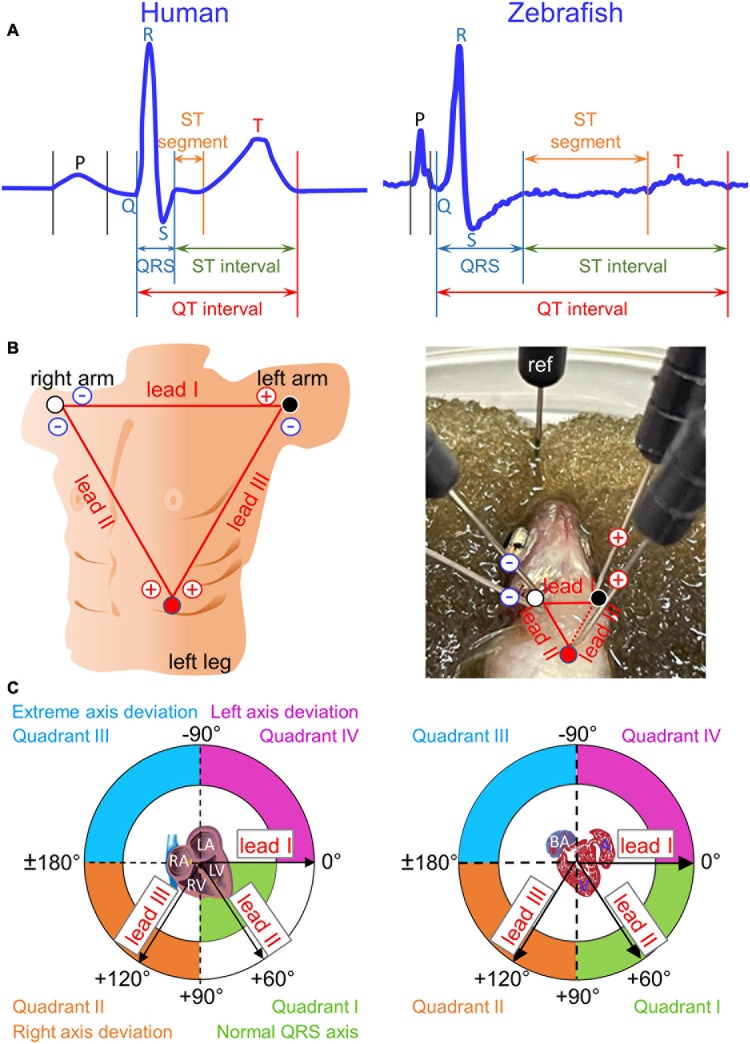
Einthoven’s triangle and Cabrera circle. **(A)** Definitions of durations for waves (P, Q, R, S, and T), intervals (QRS, QT, and ST), and segments (ST) are illustrated in two magnified and stretched normal cardiac cycles from human lead II (manual tracing over actual recording) and from zebrafish lead reverse II (rII). The normal physiological Q wave is typically absent or barely visible in adult human lead II and adult zebrafish lead rII. **(B)** To construct human Einthoven’s triangle, three pairs of electrodes for three bipolar Einthoven leads are placed on both arms and the left leg to represent the three sides of an imaginary inverted equilateral triangle enclosing the human heart. To construct zebrafish Einthoven’s triangle, we inserted two pairs of electrodes for the two bipolar Einthoven leads, such as leads I and II (solid lines). Since all three Einthoven leads relate by Einthoven’s law (II = I + III), the *Cardiac Axis Extension* algorithm (ADInstruments) derived the remaining lead III (dashed line). For grounding, we inserted the fifth electrode, the reference (ref) electrode, either in the damp sponge or in the zebrafish anal region. **(C)** We numbered the four 90°-quadrants of the Cabrera circle clockwise, roughly corresponding to human “normal QRS axis,” “right axis deviation,” “extreme axis deviation,” and “left axis deviation.” The human QRS axis defines the main heart axis, which normally averages +60° but the range of normal values is wide, encompassing from –30° to +105°, spilling from Quadrant I onto Quadrants II and IV. The zebrafish electrical heart axes and main heart axis were not known prior to our study. RA/LA, right/left atrium; RV/LV, right/left ventricle; BA, bulbus arteriosus; A, atrium; V, ventricle.

In the practice of *in vivo* surface electrocardiogram (ECG) recording for adult zebrafish, only a single lead, either bipolar or unipolar, is used due to the physical limitation of the zebrafish chest size. A bipolar ECG lead consists of a pair of electrodes of opposite (positive and negative) polarities whereas a unipolar ECG lead consists of a single positive electrode. In either case, an additional electrode, the reference electrode, is necessary for grounding purpose. In bipolar single-lead ECG recording for adult zebrafish, the conventional approach is to position the positive electrode cranial to the heart and the negative electrode caudal to the heart (Milan et al., 2006; Sun et al., 2009; Liu et al., 2016; Lin et al., 2018; Zhao et al., 2019, 2020). Thus, the lead axis orients along the heart longitudinal axis, between −90° and −120°. We referred to this zebrafish standard bipolar lead as “reverse II” (rII) (Zhao et al., 2019) because it aligns with the human standard bipolar limb lead II (which orients at +60°), but orients in reverse direction (at roughly −120°) (Figure 1A). Apparently, the rationale for this conventional practice is to align polarities of zebrafish and human ECG components (P, QRS, and T) to ease comparison. However, this practice may lead to the erroneous inference that the cardiac electrical vectors of the two species are similar, which in turn leads to misinterpretation of the clinical relevance of the adult zebrafish heart as a model for human cardiac electrophysiology and arrhythmia studies.

Importantly, despite the popular use of zebrafish as a model for studies of human cardiac electrophysiology and cardiomyopathies, a critical gap of knowledge in the field remains regarding zebrafish electrical heart axes. Among the electrical axes of the three principal ECG components (P, QRS, and T), the QRS electrical axis defines the main axis of the heart because QRS is the most dominant of the three ECG components (Surawicz and Knilans, 2008). The term “electrical axis” of a given ECG component refers to its mean manifest electrical potential in the chest space (Criteria Committee, New York Heart Association, 1964). Although the chest space can be organized into three orthogonal planes—frontal, sagittal, and transverse, the frontal plane is the most clinically relevant for electrical heart axes due to its highest diagnostic utility (Ferrer, 1972; Surawicz and Knilans, 2008). Therefore, electrical heart axis in a given plane cannot be determined by ECG recording from a single lead (Liu et al., 2016; Zhao et al., 2020), but by *simultaneous* recording from two bipolar leads that define the same plane (Dieuaide, 1921; Criteria Committee, New York Heart Association, 1964; Surawicz and Knilans, 2008). Our aim in this study is to fill this critical gap of knowledge by defining for the first time the three electrical heart axes in the frontal plane of live normal wild-type adult zebrafish. Specifically, we introduce the bipolar dual-lead ECG recording method to adult zebrafish to “reverse translate” an established clinical strategy for determining human frontal electrical heart axes based on Einthoven’s triangle and the hexaxial Cabrera reference system (Lam et al., 2015).

The human ECG discovered 120 years ago has remained virtually unchanged to date as the gold standard technique used in routine clinical practice. Regarded as the father of modern electrocardiography, Willem Einthoven was awarded the 1924 Nobel Prize in Physiology or Medicine for “his discovery of the mechanism of the (human) ECG” (Zetterström, 2009). He coined the term ECG (1893), invented the first practical ECG recording device (1903), and designed the three standard bipolar limb leads in the frontal plane− I, II, and III−in constructing Einthoven’s triangle (1912). The use of these three Einthoven leads has since become universally entrenched in clinical ECG practice. These three Einthoven leads, placed on both arms and the left leg, represent the three sides of an imaginary inverted equilateral triangle in the frontal plane enclosing the human heart (Figure 1B). In this closed circuit, the three leads relate according to Einthoven’s Law, which states that voltage in lead II is the sum of voltages in leads I and III (Einthoven, 1906; Dieuaide, 1921; Einthoven et al., 1950; Surawicz and Knilans, 2008). Thus, by exploiting this relation, we can construct zebrafish Einthoven’s triangle robustly and reproducibly by simultaneous recording from just any two (instead of all three) Einthoven leads, such as lead I (orienting along the heart horizontal axis) and lead II or III (orienting along the heart longitudinal axis).

## Materials and Methods

We conducted this study with approval by the UCLA Institutional Animal Care and Use Committee in accordance with the US National Institutes of Health *Guide for the Care and Use of Laboratory Animals*.

### Determination of Electrical Heart Axes in the Frontal Plane

The voltage amplitude and polarity of an ECG component registered in a given lead corresponds to the length and direction of the cardiac vector projection onto that lead axis. However, electrical heart axis of a given ECG component in a given plane cannot be determined from a single lead (Liu et al., 2016; Zhao et al., 2020). Instead, it requires simultaneous recording of that ECG component from at least two bipolar leads in the same plane during the same cardiac cycle (Criteria Committee, New York Heart Association, 1964; Ferrer, 1972; Surawicz and Knilans, 2008). Thus, electrical axis in the frontal plane is the vector sum of at least two cardiac vector projections onto two Einthoven lead axes, which define the frontal plane (Dieuaide, 1921). The direction of electrical axis in the frontal plane is expressed in terms of an angle (angle α) that it makes with the horizontal, which is also lead-I axis or 0° in the Cabrera system (Figure 1C; Dieuaide, 1921; Proger and Davis, 1930; Ferrer, 1972; Surawicz and Knilans, 2008).

In the ECG method, “unipolar” and “bipolar” refer to the number of electrodes of a given lead, exclusive of the reference electrode: one positive electrode for a unipolar lead and one pair of positive and negative electrodes for a bipolar lead. Unipolar single-lead ECG recording (an earlier practice in adult zebrafish ECG recording) requires insertion of a single positive electrode into the chest. Bipolar single-lead ECG recording (the most common and current practice for adult zebrafish ECG recording) requires insertion of a pair of electrodes (one positive, one negative) into two positions in the chest that are cranial and caudal to the heart. In contrast, as illustrated in Figure 1B, *right panel*, bipolar dual-lead ECG recording (which to our knowledge has never been attempted in zebrafish to date) requires insertion of two pairs of electrodes (two positives and two negatives) into three positions in the chest: right of ventricular base, left of ventricular base, and ventricular apex. Of note, Figure 1B *right panel* illustrates the basic but important concept that one electrode can be dedicated to only one lead at a time. For example, at the position of the right of the ventricular base, lead I and lead II cannot share a common negative electrode because each of these two leads requires its own separate negative electrode. Therefore, bipolar triple-lead ECG recording to construct Einthoven’s triangle as done in humans would require insertion of three pairs of electrodes (three positives and three negatives) into three positions in the chest: right arm, left arm, and left leg (Figure 1B, *left panel*). At present, simultaneous bipolar triple-lead ECG recording is not feasible for even the largest among adult zebrafish because their small chest cannot accommodate or tolerate such amount of hardware from current commercially available ECG data acquisition systems.

Fortunately, because all three Einthoven leads relate by Einthoven’s law (II = I + III) (Einthoven, 1906; Dieuaide, 1921; Surawicz and Knilans, 2008), we exploited this relation to construct zebrafish Einthoven’s triangle robustly and reproducibly by simultaneous recording from just two leads, such as leads I and II. In other words, while it is critical to record from two Einthoven leads simultaneously to determine a frontal electrical heart axis, it is not necessary to record from all three Einthoven leads for four reasons. First, as mentioned above, zebrafish cannot tolerate simultaneous ECG recording from three bipolar leads. Second, bipolar triple-lead ECG is unnecessary because construction of Einthoven’s triangle and calculation of electrical heart axes in the frontal plane requires simultaneous recording from just two Einthoven leads (Proger and Davis, 1930; Ferrer, 1972; Surawicz and Knilans, 2008). Third, if needed for other purposes, advanced software allows robust derivation of simultaneous data from the third unrecorded Einthoven lead using simultaneous recordings from the other two Einthoven leads. Lastly, we confirmed that in this study of normal subjects, manual calculations of a normal frontal electrical axis yielded similar result, whether by summing the vector projection on recorded lead I with that on recorded lead II or with that on derived lead III.

In summary, we calculated the electrical axis in the frontal plane of a given ECG component (P, QRS, or T) as the vector sum of two vector projections of that electrical activity during the same cardiac cycle on lead-I and lead-II axes in the Cabrera system (Kligfield et al., 2007; Figure 1C). For ease of reference, we numbered the four 90°-quadrants of the Cabrera system clockwise. The quadrants roughly correspond to human QRS axis (or main axis) in “normal axis” (Quadrant I, between 0° and +90°), “right axis deviation” (Quadrant II, between +90° and ± 180°), “extreme axis deviation” (Quadrant III, between ±180° and −90°), and “left axis deviation” (Quadrant IV, between −90° and 0°). We calculated all human and zebrafish electrical heart axes by two methods: manually (by summing leads I and II) and automatically (by using software). Our manual calculations confirmed the accuracies of software calculations in the twelve-lead ECG reports for humans and in the LabChart six-frontal-lead ECG reports for zebrafish (see section “Data Analysis”).

### *In vivo* ECG

We constructed 24 human Einthoven’s triangles using Einthoven’s-lead data from normal twelve-lead ECGs (*n* = 12M, 12F) of de-identified (18–70-year-old) adults. We constructed 30 zebrafish Einthoven’s triangles from healthy adult (12–18-month-old) wild-type AB zebrafish (*n* = 15M, 15F; 400–600 mg) from the UCLA Zebrafish Core Laboratory.

For zebrafish *in vivo* ECG recording, following immobilization by tricaine (0.02%; ≤3-min), we transferred zebrafish to a damp sponge submerged in fish water and positioned zebrafish ventral surface up for the insertion of five 29-gauge stainless steel electrodes (Figure 1A) to construct an inverted equilateral Einthoven’s triangle as follows:

• Both negative electrodes at the ventricular base, in the ventral midline 1–2 mm above the operculum lower edges.
• Lead-I positive electrode at the ventricular base, 2-mm left laterally to lead-I negative electrode.
• Lead-II positive electrode at the ventricular apex, 1-mm left laterally and 2-mm caudally to lead-II negative electrode.
• The reference electrode near the anus or on the damp sponge next to the zebrafish.

We recorded bipolar dual-lead ECG simultaneously from leads I and II at room temperature using PowerLab 4/35 data acquisition system and Dual Bio Amp FE 232 amplifier (ADInstruments). As previously reported (Zhao et al., 2019), we achieved satisfactory signal-to-noise ratio with the recording setting of 2 mV, low pass of 120 Hz, and high pass of 0.03 s. Upon study completion, we euthanized zebrafish by 2–4°C ice water submersion.

### Data Analysis

**FIGURE 2:**
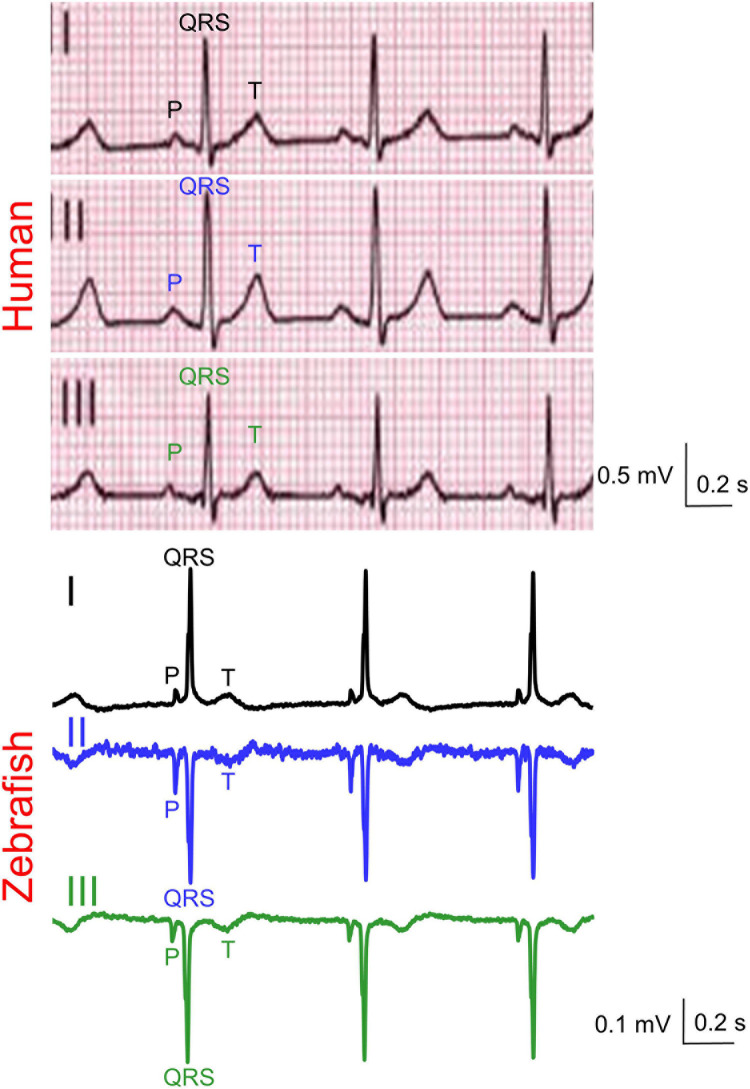
Representative simultaneous Einthoven ECG recordings from an adult man and an adult male zebrafish. Zebrafish lead III was software-derived based on Einthoven’s Law (II = I + III).

We analyzed data using LabChart Pro (ADInstruments). We performed ECG data analysis as previously reported (Zhao et al., 2019). Briefly, we verified the accuracy of LabChart auto-identification of all waveforms and corrected rare auto-identification errors (which could occur when the P wave amplitude occasionally far exceeded the R wave amplitude). Regarding durations, we followed all conventional definitions of durations for waves (P, Q, R, S, and T), intervals (QRS, QT, and ST), and segments (ST) (Figure 1A). Theoretically, the QRS complex comprises of three waves: the Q, R, and S waves and the QRS duration covers from the start of the Q wave to the end of the S wave. However, because not all three waves are always present in a given lead (true for both humans and zebrafish), the duration of the QRS complex in that lead may be reduced to the duration(s) of just one (or two) of the three waveforms actually inscribed (Figure 2). The QT interval comprises the QRS complex, ST segment, and T wave. The ST interval (covering from the end of the QRS complex to the end of the T wave) comprises the ST segment (covering from the end of the QRS complex to the start of the T wave) and the T wave: ST interval = ST segment + T-wave duration (Figure 1A). As previously reported (Zhao et al., 2019), we corrected the QT interval (QTc) for heart rate (denoted by RR interval) using Bazett’s formula: $Q\,T\,c=Q\,T÷\sqrt{R\,R}$. Likewise, we corrected the ST segment (STc) for heart rate $S\,T\,c=S\,T÷\sqrt{R\,R}$.

Regarding amplitudes, we followed the conventional practice of measuring wave amplitude (P, Q, R, S, and T) from the isoelectric baseline to the wave peak. By definition, the Q wave is the first negative deflection of the QRS complex whereas the R wave is the first positive deflection of the QRS complex. The S wave is the negative deflection of the QRS complex that follows the R wave. If the initial QRS vector is directed away from the positive electrode of a lead, then a Q wave is inscribed. Conversely, if the initial QRS vector is directed toward the positive electrode of a lead, then an R wave is inscribed. We determined the QRS net amplitude and polarity by R – (Q+S).

As a bonus, although not at all needed for the construction of Einthoven’s triangle or for the calculation of any frontal electrical heart axis, the *Cardiac Axis Extension* algorithm (ADInstruments) can use data from simultaneous recordings of leads I-II to derive information of the four remaining unrecorded frontal leads during the same cardiac cycles. Those four frontal leads include Einthoven bipolar lead III and three unipolar augmented limb leads (aV_R_, aV_L_, and aV_F_, with “R,” “L,” “F,” and “a” short for “right,” “left,” “foot,” and “augmented,” respectively). As mentioned above, we confirmed experimentally the reliability of the derivation algorithm for lead III. For the same cardiac cycle in the same zebrafish, we obtained the same electrical-axis result whether by summing the two vectors from recorded lead I and derived lead III or by summing the two vectors from recorded lead I and recorded lead II.

### Statistical Analysis

We analyzed statistics using GraphPad Prism. We expressed categorical variables as percent (%) and continuous variables as mean ± SD, median, first and third quartiles, minimum and maximum. We evaluated data distribution normality using the Shapiro-Wilk test (Whitley and Ball, 2002). We evaluated statistical significance of differences using *P* < 0.05 and (for continuous variables) 95% confidence interval (95% CI) excluding the null value as standards of significance. We estimated the (95% CI) using the bootstrap-resampling method with 10,000 replications (Manly, 2007). For continuous variables, we compared lead differences (for the same subject) using the Wilcoxon paired signed rank test, species differences (for the same lead) and sex differences (for the same species and lead) using the Mann-Whitney-Wilcoxon test. For categorical variables, we used Fisher’s exact test.

## Results

**FIGURE 3:**
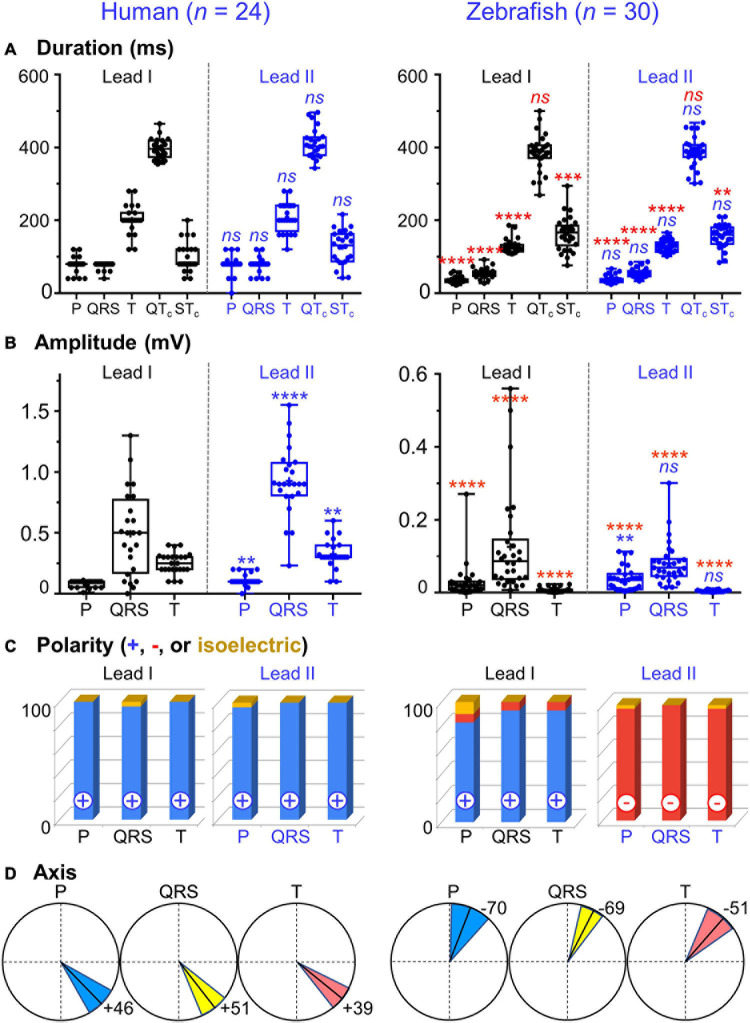
Lead- and species-comparison. **(A)** No lead differences existed in the durations of any waveforms for either species. However, for both leads, corrected QT (QTc) interval was the only waveform of similar duration in both species and corrected ST (STc) segment was the only waveform longer in zebrafish than in humans. **(B)** Lead II consistently registered larger amplitudes than lead I for all three human ECG waveforms, but only for the zebrafish P-wave. Not surprisingly, all ECG voltages were smaller in zebrafish than in humans. **(C)** The P, QRS, and T polarities were predominantly positive in both human leads I-II and zebrafish lead I, but negative in zebrafish lead II. Consequently, T-wave concordance was positive in human leads I-II and zebrafish lead I, but negative in zebrafish lead II. **(D)** The human and zebrafish cardiac electrical axes in the frontal plane are reflections across 0° in the Cabrera system, in Quadrants I and IV, respectively. In both species, the dominant QRS axis defined the main heart axis. Symbols: **(A,B)** Sample sizes: Human (*n* = 24), zebrafish (*n* = 30). Box-plot bar, mean; whiskers, SD; circles, subjects. *P*-values in blue for lead comparison of the same subject (Wilcoxon paired signed rank test), in red for species comparison of the same lead (Mann-Whitney-Wilcoxon test). *^*ns*^P* > 0.05, **P < 0.01, ***P < 0.001, ****P < 0.0001. **(D)** Line, mean; wedge, (95% CI).

Zebrafish (*n* ≥ 30) and humans (*n* = 24) displayed sinus rhythm at baseline (Figure 2). Results for amplitudes, durations, polarities, and heart axes are illustrated in Figures 3A–D and reported in Tables 1–4, respectively.

**TABLE 1:** Duration of ECG components on Einthoven leads in zebrafish vs. humans.

**Duration (ms)**	**P wave Mean ± SD**	**QRS complex Mean ± SD**	**T wave Mean ± SD**	**QTc interval Mean ± SD**	**STc segment Mean ± SD**
**Zebrafish lead I**					
Total (*n* = 30)	35 ± 10	55 ± 19	127 ± 20	385 ± 49	163 ± 45
M (*n* = 15)	30 ± 6	51 ± 14	136 ± 24	373 ± 54	154 ± 47
F (*n* = 15)	40 ± 10	58 ± 11	119 ± 7	398 ± 41	173 ± 42
Zebrafish M vs. F lead I *P*-value, [95% CI]	***0.003* [3,15]**	*0.07* [1,16]	***0.02* [−24,−2]**	*0.4* [−16,71]	*0.3* [−20,55]
**Zebrafish lead II**					
Total (*n* = 30)	37 ± 11	56 ± 13	128 ± 16	387 ± 41	156 ± 31
M (*n* = 15)	34 ± 10	52 ± 13	136 ± 16	378 ± 48	152 ± 33
F (*n* = 15)	40 ± 10	60 ± 9	119 ± 11	396 ± 32	161 ± 29
Zebrafish M vs. F lead II *P*-value, [95% CI]	*0.05* [0,13]	***0.03* [4,16]**	***0.004* [−29,−5]**	*0.2* [−11,51]	*0.4* [−14,33]
Zebrafish lead I vs. II *P*-value, [95% CI]	*0.09* [0,3]	*0.39* [−1,2]	*0.62* [−4,4]	*0.32* [0,7]	*0.84* [−14,6]
**Human lead I**					
Total (*n* = 24)	78 ± 22	76 ± 10	203 ± 39	397 ± 28	119 ± 35
M (*n* = 12)	87 ± 21	80 ± 0	213 ± 27	395 ± 28	102 ± 24
F (*n* = 12)	68 ± 18	72 ± 13	192 ± 46	399 ± 29	136 ± 37
Human M vs. F lead I *P*-value, [95% CI]	***0.04* [−32,−5]**	*0.09* [−20,0]	*0.2* [−60,0]	*0.9* [−22,30]	***0.02*** **[3,65]**
**Human lead II**					
Total (*n* = 24)	79 ± 25	77 ± 21	209 ± 41	412 ± 40	125 ± 47
M (*n* = 12)	88 ± 16	80 ± 19	228 ± 30	421 ± 45	113 ± 56
F (*n* = 12)	78 ± 21	74 ± 24	190 ± 42	402 ± 34	138 ± 33
Human M vs. F lead II *P*-value, [95% CI]	*0.6* [−40,0]	*0.6* [−30,0]	***0.04* [−80,−1]**	*0.4* [−51,19]	*0.2* [−16,71]
Human lead I vs. II *P*-value, [95% CI]	*0.1* [−10,13]	*0.1* [−7,9]	*0.09* [−13,27]	*0.07* [−1,30]	*0.4* [−14,28]
Fish vs. human lead I *P*-value, [95% CI]	***<0.0001* [42,50]**	***<0.0001* [19,28]**	***<0.0001* [68,89]**	*0.3* [−11,29]	***0.0002* [−68,−21]**
Fish vs. human lead II *P*-value, [95% CI]	***<0.0001* [42,50]**	***<0.0001* [18,31]**	***<0.0001* [65,103]**	*0.06*, [−2,40]	***0.01* [−52,−5]**

*We compared lead differences (for the same subject) using the Wilcoxon paired signed rank test, sex differences (for the same species and lead) and species differences (for the same lead) using the Mann-Whitney-Wilcoxon test. We estimated *P*-value and 95% confidence interval [95% CI] by bootstrapping with 10,000 replications. P-values are shown in italics; statistically significant P-values and [95%CI] in bold font. QTc, corrected QT interval; STc, corrected ST segment.*

**TABLE 2:** Amplitude of ECG components on Einthoven leads in zebrafish vs. humans.

**Amplitude (mV)**	**P wave Mean ± SD**	**QRS complex Mean ± SD**	**T wave Mean ± SD**	**P:QRS:T**
**Zebrafish lead I**				
Total (*n* = 30)	0.028 ± 0.049	0.126 ± 0.049	0.006 ± 0.006	0.36:1.00:0.15
M (*n* = 15)	0.016 ± 0.011	0.080 ± 0.011	0.006 ± 0.007	0.29:1.00:0.11
F (*n* = 15)	0.041 ± 0.067	0.172 ± 0.178	0.008 ± 0.007	0.42:1.00:0.19
Zebrafish M vs. F lead I *P*-value [95% CI]	*0.4* [−0.007,0.025]	*0.3* [0.017,0.128]	*0.3*, [−0.002,0.005]	
**Zebrafish lead II**				
Total (*n* = 30)	0.037 ± 0.032	0.081 ± 0.059	0.004 ± 0.003	0.55:1.00:0.08
M (*n* = 15)	0.046 ± 0.039	0.098 ± 0.072	0.005 ± 0.003	0.57:1.00:0.09
F (*n* = 15)	0.028 ± 0.020	0.063 ± 0.037	0.004 ± 0.002	0.52:1.00:0.08
Zebrafish M vs. F lead II *P*-value [95%CI]	*0.2* [−0.034,0.006]	*0.1* [−0.054,0.008]	*0.5* [−0.004,0.001]	
Zebrafish lead I vs. II *P*-value [95% CI]	***0.008* [0.002,0.020]**	*0.6* [−0.100,0.015]	*0.08* [−0.004,0.001]	
**Human lead I**				
Total (*n* = 24)	0.08 ± 0.03	0.49 ± 0.35	0.25 ± 0.09	0.16:1.00:0.51
M (*n* = 12)	0.09 ± 0.02	0.47 ± 0.34	0.28 ± 0.09	0.19:1.00:0.60
F (*n* = 12)	0.08 ± 0.03	0.51 ± 0.37	0.22 ± 0.07	0.16:1.00:0.43
Human M vs. F lead I *P*-value [95% CI]	*0.2* [−0.05,0.00]	*0.8* [−0.3,0.4]	*0.08* [−0.11,0.00]	
**Human lead II**				
Total (*n* = 24)	0.11 ± 0.05	0.93 ± 0.28	0.33 ± 0.11	0.12:1.00:0.35
M (*n* = 12)	0.12 ± 0.05	0.90 ± 0.34	0.38 ± 0.11	0.13:1.00:0.42
F (*n* = 12)	0.10 ± 0.03	0.95 ± 0.23	0.28 ± 0.10	0.11:1.00:0.29
Human M vs. F lead II *P*-value [95% CI]	*0.3* [−0.05,0.00]	*0.6* [−0.18,0.29]	*0.1* [−0.2,0.0]	
Human lead I vs. II *P*-value [95% CI]	***0.004* [0.006,0.050]**	***<0.0001* [0.12,0.80]**	***0.0009* [0.03,0.12]**	
Fish vs. human lead I *P*-value [95% CI]	***<0.0001* [0.05,0.08]**	***<0.0001* [0.35,0.64]**	***<0.0001* [0.21,0.29]**	
Fish vs. human lead II *P*-value [95% CI]	***<0.0001* [0.06,0.09]**	***<0.0001* [0.78, 0.91]**	***<0.0001* [0.296,0.300]**	

*We compared lead differences (for the same subject) using the Wilcoxon paired signed rank test, sex differences (for the same species and lead) and species differences (for the same lead) using the Mann-Whitney-Wilcoxon test. We estimated *P*-value and 95% confidence interval [95% CI] by bootstrapping with 10,000 replications. P-values are shown in italics; statistically significant *P*-values and [95%CI] in bold font.*

**TABLE 3:** Polarity of ECG components on Einthoven leads in zebrafish vs. humans.

**Polarity**	**P wave *n*/*n*_total_ (%)**	**QRS complex *n*/*n*_total_ (%)**	**T wave *n*/*n*_total_ (%)**	**QRS-T concordance *n*/*n*_total_ (%)**
**Zebrafish lead I**				
Total (*n* = 30)	(+): 25/30 (83%) (−): 2/30 (7%) (0): 3/30 (10%)	(+): 28/30 (93%) (−): 2/30 (7%) (0): 0/30 (0%)	(+): 28/30 (93%) (−): 2/30 (7%) (0): 0/30 (0%)	(+): 25/30 (83%) (−): 2/30 (7%) (0): 3/30 (10%)
M (*n* = 15)	(+): 12/15 (80%) (−): 1/15 (7%) (0): 2/15 (13%)	(+): 14/15 (93%) (−): 1/15 (7%) (0): 0/15 (0%)	(+): 14/15 (93%) (−): 1/15 (7%) (0): 0/15 (0%)	(+): 12/15 (80%) (−): 1/15 (7%) (0): 2/15 (13%)
F (*n* = 15)	(+): 13/15 (87%) (−): 1/15 (7%) (0): 1/15 (7%)	(+): 14/15 (93%) (−): 1/15 (7%) (0): 0/15 (0%)	(+): 14/15 (93%) (−): 1/15 (7%) (0): 0/15 (0%)	(+):14/15 (93%) (−):1/15 (7%) (0): 0/15 (0%)
Zebrafish M vs. F lead I	*P > 0.99*	*P > 0.99*	*P > 0.99*	*P = 0.59*
**Zebrafish lead II**				
Total (*n* = 30)	(+): 0/30 (0%) (−): 29/30 (97%) (0): 1/30 (3%)	(+): 0/30 (0%) (−): 30/30 (100%) (0): 0/30 (0%)	(+): 0/30 (0%) (−): 29/30 (97%) (0): 1/30 (3%)	(+): 0/30 (0%) (−): 28/30 (93%) (0): 2/30 (7%)
M (*n* = 15)	(+): 0/15 (0%) (−): 14/15 (93%) (0): 1/15 (7%)	(+): 0/15 (0%) (−): 15/15 (100%) (0): 0/15 (0%)	(+): 0/15 (0%) (−): 14/15 (93%) (0): 1/15 (7%)	(+): 0/15 (0%) (−): 13/15 (87%) (0): 2/15 (13%)
F (*n* = 15)	(+): 0/15 (0%) (−): 15/15 (100%) (0): 0/15 (0%)	(+): 0/15 (0%) (−): 15/15 (100%) (0): 0/15 (0%)	(+): 0/15 (0%) (−): 15/15 (100%) (0): 0/15 (0%)	(+): 0/15 (0%) (−): 15/15 (100%) (0): 0/15 (0%)
Zebrafish M vs. F lead II	*P > 0.99*	*P > 0.99*	*P > 0.99*	*P = 0.481*
Zebrafish lead I vs. II	***P < 0.0001***	***P < 0.0001***	***P < 0.0001***	***P < 0.0001***
**Human lead I**				
Total (*n* = 24)	(+): 24/24 (100%) (−): 0/24 (0%) (0): 0/24 (0%)	(+): 23/24 (96%) (−): 0/24 (0%) (0): 1/24 (4%)	(+): 24/24 (100%) (−): 0/24 (0%) (0): 0/24 (0%)	(+): 23/24 (96%) (−): 0/24 (0%) Undetermined: 1/24 (4%) (0): 0/24 (0%)
M (*n* = 12)	(+): 12/12 (100%) (−): 0/12 (0%) (0): 0/12 (0%)	(+):12/12 (100%) (−): 0/12 (0%) (0): 0/12 (0%)	(+):12/12 (100%) (−): 0/12 (0%) (0): 0/12 (0%)	(+): 12/12 (100%) (−): 0/12 (0%) (0): 0/12 (0%)
F (*n* = 12)	(+): 12/12 (100%) (−): 0/12 (0%) (0): 0/12 (0%)	(+): 11/12 (92%) (−): 0/12 (0%) (0): 1/12 (8%)	(+): 12/12 (100%) (−): 0/12 (0%) (0): 0/12 (0%)	(+): 11/12 (92%) (−): 0/12 (0%) Undetermined: 1/12 (8%) (0): 0/12 (0%)
Human M vs. F lead I	*P > 0.99*	*P > 0.99*	*P > 0.99*	*P > 0.99*
**Human lead II**				
Total (*n* = 24)	(+): 23/24 (96%) (−): 0/24 (0%) (0): 1/24	(+): 24/24 (100%) (−): 0/24 (0%) (0): 0/24 (0%)	(+): 24/24 (100%) (−): 0/24 (0%) (0): 0/24 (0%)	(+): 24/24 (100%) (−): 0/24 (0%) (0): 0/24 (0%)
M (*n* = 12)	(+):11/12 (92%) (−): 0/12 (0%) (0): 1/12 (8%)	(+):12/12 (100%) (−): 0/12 (0%) (0): 0/12 (0%)	(+):12/12 (100%) (−): 0/12 (0%) (0): 0/12 (0%)	(+): 12/12 (100%) (−): 0/12 (0%) (0): 0/12 (0%)
F (*n* = 12)	(+): 12/12 (100%) (−): 0/12 (0%) (0): 0/12 (0%)	(+): 12/12 (100%) (−): 0/12 (0%) (0): 0/12 (0%)	(+): 12/12 (100%) (−): 0/12 (0%) (0): 0/12 (0%)	(+): 12/12 (100%) (−): 0/12 (0%) (0): 0/12 (0%)
Human M vs. F lead II	*P > 0.99*	*P > 0.99*	*P > 0.99*	*P > 0.99*
Human lead I vs. II	*P > 0.99*	*P > 0.99*	*P > 0.99*	*P > 0.99*
Fish vs. human lead I	*P = 0.06*	*P > 0.99*	*P = 0.5*	*P = 0.2*
Fish vs. human lead II	***P < 0.0001***	***P < 0.0001***	***P < 0.0001***	***P < 0.0001***

*We compared differences between leads, species, and sexes using Fisher’s exact test. P-values are shown in italics; statistically significant P-values in bold font. Polarity (0), isoelectric; QRS-T concordance (0), discordance.*

**TABLE 4:** Electrical heart axes in the frontal plane of zebrafish vs. humans.

**Vector axis (^*o*^)**	**Mean ± SD; Range** (**95% CI**)	**Dominant quadrant (% subjects)**
**Zebrafish P axis**		
Total (*n* = 30)	−70 ± 49; (−156,142) [−89,−51]	IV (80%)
M (*n* = 15)	−86 ± 31; (−156,−46) [−103,−69]	IV (73%)
F (*n* = 15)	−54 ± 60; (−140,142) [−87,−21]	IV (87%)
**Zebrafish QRS axis (main heart axis)**		
Total (*n* = 30)	−69 ± 25; (−167,−40) [−78,−60]	IV (93%)
M (*n* = 15)	−72 ± 28; (−167,−45) [−88,−56]	IV (93%)
F (*n* = 15)	−66 ± 21; (−130,−40) [−78,−55]	IV (93%)
**Zebrafish T axis**		
Total (*n* = 30)	−51 ± 49; (−136,150) [−71,−33]	IV (83%)
M (*n* = 15)	−55 ± 34; (−136,−9) [−74,−36]	IV (87%)
F (*n* = 15)	−47 ± 62; (−122,150) [−82,−13]	IV (80%)
**Human P axis**		
Total (*n* = 24)	+46 ± 24; (−4,84) [35,56]	I (92%)
M (*n* = 12)	+49 ± 25; (−4,73) [32,65]	I (92%)
F (*n* = 12)	+43 ± 24; (−1,84) [27,58]	I (92%)
**Human QRS axis (main heart axis)**		
Total (*n* = 24)	+51 ± 27; (5,91) [40,62]	I (100%)
M (*n* = 12)	+56 ± 26; (5,91) [39,72]	I (100%)
F (*n* = 12)	+46 ± 28; (8,90) [29,64]	I (100%)
**Human T axis**		
Total (*n* = 24)	+39 ± 24; (−30,71) [29,49]	I (92%)
M (*n* = 12)	+46 ± 12; (25,75) [39,54]	I (100%)
F (*n* = 12)	+32 ± 30; (−30,71) [13,52]	I (83%)
	***P*-value**	**[95% CI]**
Zebrafish M vs. F P axis	*0.06*	[−1,47]
Zebrafish M vs. F QRS axis	*0.4*	[−8,16]
Zebrafish M vs. F T axis	*0.9*	[−28,24]
Human M vs. F P axis	*0.3*	[−26,10]
Human M vs. F QRS axis	*0.5*	[−35,14]
Human M vs. F T axis	*0.3*	[−30,6]
Fish vs. Human P axis	***<0.0001***	**[107,304]**
Fish vs. Human QRS axis	***<0.0001***	**[104,133]**
Fish vs. Human T axis	***<0.0001***	**[81,109]**

*We compared differences between species and sexes using the Mann-Whitney-Wilcoxon test. P-values are shown in italics; statistically significant P-values and 95% confidence intervals [95% CI] in bold font.*

### P Wave

The P wave reflects atrial activation (excitation or depolarization). The P wave morphology is generated by a single dipole that reflects a simple pattern of atrial activation sequence. In humans, atrial activation begins in the sinoatrial node and spreads radially to depolarize the right atrium, the interatrial septum, then the left atrium. In this study, no lead difference existed in human P-wave duration (lead-I 78 ± 22 ms vs. lead-II 79 ± 25 ms; Figure 3A and Table 1). However, human P wave was taller in lead-II (0.11 ± 0.05 mV) than lead-I (0.08 ± 0.03 mV; *P* = 0.004 [0.006,0.050]; Figure 3B and Table 2). Human P wave was the smallest of all three ECG components (12–16% of QRS amplitude). It was predominantly upright in both leads (lead-I 100%, lead-II 96%; Figure 3C and Table 3). Therefore, human P-wave axis fell mostly within Quadrant I (92%, +46 ± 24° [35,56]; Figure 3D and Table 4). Human P-wave sex difference existed only in lead-I duration with male P-wave duration being longer (M 87 ± 21 ms > F 68 ± 18 ms, *P* = 0.04 [−32,−5]).

In zebrafish, atrial activation begins in the sinoatrial node located in the sinus venosus and spreads radially to depolarize the atrium (Sedmera et al., 2003). In this study, no lead difference existed in zebrafish P-wave duration (lead-I 35 ± 10 ms vs. lead-II 37 ± 11 ms; Figure 3A and Table 1). Thus, the P-wave duration, reflecting atrial activation, was ∼55% shorter in zebrafish than in humans (*P <* 0.0001 [42,50] for both leads I and II). Like human P wave, zebrafish P wave was taller in lead-II (0.037 ± 0.032 mV) than lead-I (0.028 ± 0.049 mV; *P* = 0.008 [0.002,0.020]; Figure 3B and Table 2). However, at 36–55% of QRS amplitude, zebrafish P wave was not the smallest, but the second tallest ECG components, consistent with a larger atrial-to-ventricular size ratio in zebrafish than in humans. Not surprisingly, the P-wave amplitude was ∼65% smaller in zebrafish than in humans (lead-I *P <* 0.0001 [0.05,0.08], lead-II *P <* 0.0001 [0.06,0.09]). Like humans, zebrafish P wave was commonly upright (83%) in lead I. However, unlike humans, zebrafish P wave was predominantly inverted in lead II (97%; Figure 3C and Table 3). Therefore, zebrafish P-wave axis fell mostly within Quadrant IV (80%, −70 ± 49° [−89, −51]), a reflection of human P-wave axis across 0° in the Cabrera system (Figure 3D and Table 4). Like humans, zebrafish P-wave sex difference existed only in lead-I duration but in reverse direction with female P-wave duration being longer (F 40 ± 10 ms > M 30 ± 6 ms, *P* = 0.003 [3,15]).

### QRS Complex and Main Heart Axis

The QRS complex reflects ventricular activation (excitation or depolarization). Unlike the P wave, the complicated QRS morphology may not be readily explained by a single dipole but by residual uncanceled potentials of multiple electromotive forces propagating simultaneously in several directions. In humans, ventricular activation is divided into three phases: early, middle, and late, represented, respectively, by three vectors Q, R, and S (Surawicz and Knilans, 2008). The human Q vector directs rightward and anteriorly and, as Surawicz and Knilans cautioned, human Q wave represents more than just septal depolarization. In the normal human heart, due to the greater thickness of the left compared to the right ventricular wall and due to the relative positions of the two ventricles, the normal human R wave represents predominantly the uncanceled potentials of transmural activation of the left ventricle. The human R vector directs leftward. The S wave, or the terminal portion of the QRS complex, represents posterobasal activation of both ventricles and the septum. The human S vector directs superiorly and either anteriorly or posteriorly (Surawicz and Knilans, 2008).

In this study, no lead difference existed in human QRS duration (lead-I 76 ± 10 ms vs. lead-II 77 ± 21 ms; Figure 3A and Table 1). However, human QRS was taller in lead-II (0.93 ± 0.28 mV) than lead-I (0.49 ± 0.35 mV; *P* < 0.0001 [0.12,0.80]; Figure 3B and Table 2). Human QRS was largely upright in both leads (lead-I 96%, lead-II 100%; Figure 3C and Table 3), Thus, the QRS axis fell within Quadrant I (100%, +51 ± 27° [40,62]; Figure 3D and Table 4). Because human QRS was the tallest ECG component and was R-wave dominant, the QRS axis corresponded to the R axis and defined the human main heart axis. No human QRS sex difference existed in duration, amplitude, or axis.

In zebrafish, ventricular activation spreads from the atrioventricular ring (a functional equivalent of the human atrioventricular node) through two main trabecular bundles and the radial trabecular network (a functional equivalent of the human His-Purkinje system) to the ventricular apex (Sedmera et al., 2003), then from apex to base (Sedmera et al., 2003; Zhao et al., 2020). In this study, no lead difference existed in zebrafish QRS duration (lead-I 55 ± 19 ms vs. lead-II 56 ± 13 ms; Figure 3A and Table 1) or amplitude (lead-I 0.126 ± 0.049 mV vs. lead-II 0.081 ± 0.059 mV; Figure 3B and Table 2). The QRS duration, reflecting ventricular activation, was almost 30% shorter in zebrafish than in humans (lead-I *P <* 0.0001 [19,28], lead-II *P <* 0.0001 [18,31]). Not surprisingly, the QRS amplitude was ∼80% smaller in zebrafish than in humans (lead-I *P <* 0.0001 [0.35,0.64], lead-II *P <* 0.0001 [0.78,0.91]). Like humans, zebrafish QRS defined the main heart axis as the most dominant ECG component and was largely upright (93%) in lead I such that lead-I QRS axis corresponded to its R axis. However, unlike humans, zebrafish QRS was invariably inverted in lead II (100%; Figure 3C and Table 3), thus lead-II QRS axis corresponded to its QS axis instead. Therefore, the zebrafish main heart axis, as determined by the QRS axis, fell within Quadrant IV (93%, −69 ± 25° [−78,−60]), a reflection of the human main heart axis across 0° in the Cabrera system (Figure 3D and Table 4). Zebrafish QRS sex difference existed only in lead-II duration with female QRS duration being longer (F 60 ± 9 ms > M 52 ± 13 ms, *P* = 0.03 [4,16]).

### T Wave and Concordance With QRS

The T wave represents the uncanceled potential differences of ventricular repolarization. In humans, the normal T vector directs leftward, inferiorly, and shifted from posteriorly to anteriorly with age (Surawicz and Knilans, 2008). In this study, no lead difference existed in human T-wave duration (lead-I 203 ± 39 ms vs. lead II 209 ± 41 ms; Figure 3A and Table 1). However, human T wave was taller in lead II (0.33 ± 0.11 mV) than lead I (0.25 ± 0.09 mV, *P* = 0.0009 [0.03,0.12]; Figure 3B and Table 2). Human T wave was the second tallest ECG component (35–51% of QRS amplitude). Since it was invariably upright in both leads (both 100%; Figure 3C and Table 3), human T-wave axis fell within Quadrant I (92%, +39 ± 24° [29,49]; Figure 3D and Table 4). Human T-wave sex difference existed only in lead-II duration with male T-wave duration being longer (M 228 ± 30 ms > F 190 ± 42 ms, *P* = 0.04 [−80,−1]).

In zebrafish, no lead difference existed in T-wave duration (lead-I 127 ± 20 ms vs. lead-II 128 ± 16 ms; Figure 3A and Table 1) or amplitude (lead-I 0.006 ± 0.006 mV vs. lead-II 0.004 ± 0.003 mV; Figure 3B and Table 2). The T-wave duration, reflecting late ventricular repolarization, was ∼35% shorter in zebrafish than in humans (lead-I *P <* 0.0001 [68,89], lead-II *P <* 0.0001 [65,103]). Unlike humans, zebrafish T wave was the smallest ECG component (only 8–15% of zebrafish QRS amplitude). Not surprisingly, the T-wave amplitude was 98% smaller in zebrafish than in humans (lead-I *P <* 0.0001 [0.21,0.29], lead-II *P <* 0.0001 [0.296,0.300]). Like humans, zebrafish T wave was largely upright (93%) in lead I. However, unlike humans, zebrafish T wave was predominantly inverted in lead II (97%; Figure 3C and Table 3). Therefore, zebrafish T-wave axis fell within Quadrant IV (83%, −51 ± 49° [−71,−33]), a reflection of human T-wave axis across 0° in the Cabrera system (Figure 3D and Table 4). Like humans, zebrafish T-wave sex difference existed in durations with male T-wave duration being longer (lead-I M 136 ± 24 ms > F 119 ± 7 ms, *P* = 0.02 [−24,−2] and lead-II M 136 ± 16 ms > F 119 ± 11 ms, *P* = 0.004 [−29,−5]).

An important T-wave feature is its polarity concordance with QRS, which reflects that the sequence of ventricular repolarization is the reverse of the sequence of ventricular depolarization for both humans (Surawicz and Knilans, 2008) and zebrafish (Zhao et al., 2020). In human leads I-II, because QRS and T wave in the same lead were both positive (Figure 3C and Table 3), human T-wave concordance was positive (lead-I 96% and lead-II 100%). Like humans, zebrafish also displayed predominant T-wave concordance (lead-I 90% and lead-II 93%). However, unlike humans, zebrafish T-wave concordance was positive only in lead I (83%), but negative in lead II (93%; Figure 3C and Table 3).

### QTc Interval

By definition, the ST interval covers from the end of the QRS complex to the end of the T wave whereas the QT interval covers from the start of the QRS complex to the end of the T wave (Figure 1A). Therefore, the ST interval represents pure ventricular repolarization whereas the QT interval represents both ventricular activation and ventricular repolarization. However, in actuality, the QT interval has been firmly entrenched as a more popular surface biomarker of ventricular repolarization. Because QT prolongation or shortening may herald the emergence of potentially lethal cardiac arrhythmias, the QT interval assumes a pivotal diagnostic role.

In this study, no lead difference existed in QTc duration for humans (lead-I 397 ± 28 ms vs. lead-II 412 ± 40 ms) or zebrafish (lead-I 385 ± 49 ms vs. lead-II 387 ± 41 ms). Importantly, QTc was the only ECG waveform of similar duration between both species (lead-I *P* = 0.3 [−11,29], lead-II *P* = 0.06 [−2,40]; Figure 3A and Table 1). Because QRS and T wave were 30–35% shorter in zebrafish than in humans, zebrafish ST segment (which equals the ST interval minus the T-wave duration) must be longer than human ST segment to account for the similar QTc duration between both species. Indeed, direct measurements confirmed that the STc segment was the only waveform that was ∼30% longer in zebrafish than in humans: lead-I zebrafish 163 ± 45 vs. human 119 ± 35 ms (*P* = 0.0002 [−68,−21]); lead-II zebrafish 156 ± 31 vs. human 125 ± 47 ms (*P* = 0.01 [−52,−5]).

## Discussion

This study highlights three novel insights in the field of adult zebrafish cardiac electrophysiology:

• The first determination of the three frontal electrical heart axes, including the main heart axis, of live normal adult zebrafish using the Cabrera system derived from the first Einthoven’s triangle for normal adult zebrafish,
• The first systematic comparison of zebrafish with human Einthoven’s triangle to reveal the underlying tissue mechanisms responsible for the similarities and differences in their three frontal electrical heart axes under normal baseline, and
• The first technical breakthrough in simultaneous bipolar dual-lead ECG recording in live adult zebrafish.

### Electrical Axis and Pathophysiological Relevance of Axis Deviation

Electrical axis of the heart is a measurable vectorial quantity with both magnitude and direction. However, the direction of the human main axis (i.e., QRS axis in the frontal plane), introduced by Einthoven as angle α, has had the highest pathophysiological relevance (Proger and Davis, 1930). Indeed, determination of human P-QRS-T frontal axes has become so entrenched in routine clinical practice that to date, twelve-lead ECG data acquisition software is readily equipped to calculate all three electrical axes automatically and precisely for each recording. In this study, we found the mean main axis of normal adult humans to be +51° (Quadrant I, Figure 3D, *left panel*). Our finding is in good agreement with extensive literature reports of the mean human main axis value of +60° within the vast normal range between −30° and +105° (Proger and Davis, 1930; Hiss et al., 1960; Simonson, 1972), which encompasses the entire Cabrera Quadrant I plus two adjacent wedges in Quadrants IV and II. This extensive normal range reflects, apart from biological variation across hearts, a counterclockwise shift on the Cabrera system in the same hearts with age such that the main axis orients more inferiorly (or rightward of +90°) in childhood/early adulthood and more superiorly (or leftward of 0°) in late adulthood (Figure 1C, *left panel*).

The diagnostic significance of frontal axis deviation from the normal range (Figure 1C, *left panel*) allows clinical recognition of intraventricular conduction defects, chamber enlargement/hypertrophy, certain congenital heart defects, and even acute or chronic lung disease in the proper clinical settings (Dieuaide, 1921; Proger and Davis, 1930; Ferrer, 1972; Surawicz and Knilans, 2008; Pérez-Riera et al., 2020). For example, left axis deviation (between −30° and −90°, i.e., most of Quadrant IV) may signal acquired or congenital cardiac structural abnormalities (such as left ventricular hypertrophy, inferior myocardial infarction, ostium primum atrial septal defect, or endocardial cushion defect) or cardiac conduction abnormalities (such as left anterior fascicular block or left bundle branch block). Right axis deviation (between +90° and ± 180°, i.e., Quadrant II) may signal cardiac structural abnormalities (such as right ventricular hypertrophy or lateral myocardial infarction) or cardiac conduction or rhythm abnormalities (such as left posterior fascicular block, right bundle branch block, pre-excitation syndromes, or right ventricular outflow tract tachycardia). Interestingly, axis deviation may also signal non-cardiac etiologies. For example, left axis deviation may signal emphysema or obesity-induced diaphragm elevation whereas right axis deviation may signal chronic obstructive pulmonary disease or life-threatening acute pulmonary embolus. Even more remarkably, the clinical significance of axis deviation is not limited to just *spatial deviation* of angle α at a point in time, but also to its *temporal deviation* during the clinical course. For example, in chronic obstructive pulmonary disease, a dynamic shift of the electrical axis to the right of 30° from the patient’s former electrical axis, even without actually meeting criteria for right axis deviation, signals early cor pulmonale and impending right ventricular failure, especially when coupled with desaturation of arterial blood oxygen below 85% (Kilcoyne et al., 1970). Extreme axis deviation (between −90° and ± 180°, i.e., Quadrant III) may signal hyperkalemia, severe right ventricular hypertrophy, ventricular tachycardia, or high-risk Brugada syndrome (Pérez-Riera et al., 2020). The clinical significance of frontal axis deviation extends beyond diagnostics to prognostics. For example, left axis deviation portends higher risk of combined all-cause mortality and major adverse cardiovascular events in the three years following diagnosis of axis deviation even in the absence of ventricular conduction delay when compared to normal axis and right axis deviation (Seko et al., 2021).

In summary, normal human electrical axes in the frontal plane were well established. The diagnostic and prognostic significance of electrical frontal axis deviation were also convincingly demonstrated for humans (Dieuaide, 1921; Proger and Davis, 1930; Ferrer, 1972; Surawicz and Knilans, 2008; Pérez-Riera et al., 2020; Seko et al., 2021). As a result, this clinical significance has revolutionized modern-day clinical interpretation of the ECG to incorporate routine precise determination of all three electrical frontal axes (Ferrer, 1972).

In contrast, until our study, normal electrical axes, including the normal main axis, have not been determined for zebrafish in any of the three orthogonal planes although the method of *in vivo* surface electrocardiography for adult zebrafish was established fifteen years ago (Milan et al., 2006). To compound the uncertainty regarding zebrafish electrical axes, several sources for confusion exist regarding zebrafish ECG interpretation. By convention, the standard bipolar single lead for adult zebrafish runs along the heart longitudinal axis, but pointing to −120° (reverse II lead axis) or −90° (reverse aV_F_ lead axis) (Milan et al., 2006; Sun et al., 2009; Chaudhari et al., 2013; Liu et al., 2016; Lin et al., 2018; Zhao et al., 2019, 2020). Reversal of lead axis orientation is a common source of confusion as it may cause the misconception that zebrafish and human P-QRS-T polarities along the heart longitudinal axis align whereas they are in fact opposite (Zhao et al., 2019, 2020). Other sources of confusion include failure to discriminate between “lead” and “electrode” and inattention to electrode polarity and orientation of lead axis. The knowledge of ECG electrode polarity and orientation of lead axis provides a frame of reference critical for proper interpretation of electrical vectors and underlying tissue mechanisms, hence for translational significance. For example, QRS positivity signals that the QRS vector and underlying ventricular depolarization are both heading toward the positive electrode of that lead axis. Likewise, T-wave positivity signals that the T vector and underlying ventricular repolarization are both heading away from the positive electrode of that lead axis.

To illustrate how ECG interpretation matters for translational significance, we review a popular adult zebrafish model of apical injury (by either resection or cryoablation) as presented by Liu et al. (2016). The authors made the important discovery that adult zebrafish QRS vector reverses polarity along the heart longitudinal axis following apical injury. However, inattention to electrode polarity and orientation of lead axis led to confusion regarding the true directions of the normal vs. injured QRS vectors, hence obscuring the underlying tissue mechanisms. Using two unipolar leads apparently sharing the same frontal lead axis of −90° (reverse aV_*F*_ lead axis) placed at the ventricular apex and base, the authors found that normal QRS deflections at both base and apex are positive but QRS amplitude is larger at apex compared to base. The authors also found that following injury, the ratio of apex-to-base QRS amplitudes reverses and apical QRS deflection may become negative. Based on those QRS-amplitude ratios, the authors concluded that the normal QRS vector on the heart longitudinal axis points from “anterior” (i.e., base) to “posterior” (i.e., apex) and reverses direction to point from the injured apex to base following apical injury. In fact, the converse of the authors’ ECG interpretation is true. Normal ventricular depolarization spreads in the direction of the normal QRS vector, from apex to base, as demonstrated by many other groups, including ours (Sedmera et al., 2003; Zhao et al., 2020), and again in this study as illustrated by the negativity of QRS vector projection on lead II (Figure 3C, *left panel*). It follows that if injury reverses the QRS vector on the heart longitudinal axis, then post-injury ventricular depolarization spreads in the direction of the QRS vector of injury, from base to the injured apex.

Hence, proper ECG interpretations can reveal cardiac structure-function relationship. In the normal zebrafish heart, the electrophysiological function of ventricular depolarization from apex to base correlates logically with the structure of the two main ventricular trabeculae, known to provide direct myocardial continuity between the atrioventricular ring and the apex (Sedmera et al., 2003). Likewise, in the injured zebrafish heart, the pathophysiological reversal of ventricular depolarization from base to the injured apex also correlates logically with the structural loss of the distal, apical portions of the main trabeculae. Additionally, insight from lead I of zebrafish Einthoven’s triangle in this study can also explain why normal QRS amplitude is smaller at the base than apex as Liu et al. (2016) astutely observed. The explanation is based on the principles that ECG voltage is maximal when electrical activity travels in parallel with a lead axis and becomes isoelectric when electrical activity travels in perpendicular to a lead axis. In the normal zebrafish ventricle, once the depolarization wavefront reaches the base, it can no longer continue to propagate in parallel with the recording lead axis along the heart longitudinal axis. Instead, the depolarization wavefront switches direction to propagate along the base in parallel with the heart horizontal axis (in a left-to-right direction as revealed by QRS positivity in lead I; Figure 3C, *left panel*), thus causing the recorded longitudinal QRS voltage at the base to decrease compared to that at the apex.

On that note, Liu et al. (2016) usage of the term “cardiac vector” in reference to the QRS vector along a single-lead axis does not imply the “cardiac main axis,” which is defined by the QRS axis in the frontal plane. In this study, we made the first step toward defining the normal main axis of adult zebrafish hearts: mean −69° (Quadrant IV; Figure 3D, *right panel*), 95% CI (−60, −78), range between −40° and −167° (encompassing most of Quadrants IV and III). Thus, the main axis of normal 12–18-month-old zebrafish is a relative mirror image of the normal human main axis across the horizontal (or 0° in the Cabrera system). Had our approach of bipolar dual-lead ECG been used to calculate the QRS frontal axis for the apical injury model presented in Liu et al. (2016) study, additional insights could have been gained regarding QRS frontal axis deviation. In place of a laborious, precise calculation of angle α, it is possible to roughly determine by quick inspection of the positivity or negativity of the simultaneous QRS vector projections on two leads, such as leads I and II, the quadrant in which the main axis lies (Pardee, 1914). For example, in the case of the zebrafish apical injury model, if the QRS vector on lead-I axis does not reverse polarity following injury like the QRS vector on lead-II axis, then the injury causes left axis deviation (from Quadrant IV to Quadrant I). On the other hand, if the QRS vector on lead-I axis also reverses polarity following injury like the QRS vector on lead-II axis, then the injury causes extreme axis deviation (from Quadrant IV to Quadrant II). Therefore, knowledge of the QRS vector direction on a single-lead axis alone precludes determination of the QRS frontal axis or of QRS axis deviation.

Future large-scale longitudinal studies in normal adult zebrafish are needed not only to replicate our finding in larger populations but also to establish potential temporal axis deviation from early to late adulthood as reported in humans. More importantly, we hope that the simultaneous bipolar dual-lead ECG recording approach presented in this study will inspire routine robust assessment of adult zebrafish electrical axis deviation as an independent tool for accurate risk stratification in adult zebrafish models of cardiac conduction defects, arrhythmias, and cardiomyopathies as well as in drug screening. Which two Einthoven leads shall we record? Since modern-day software can reliably derive the remaining four frontal limb leads, it makes little difference which two Einthoven leads we choose to record. It matters more which two leads we shall measure in manual calculation of electrical axes. In this study of normal zebrafish, our two Einthoven leads of choice are leads I and II. Our choice of zebrafish lead I was straightforward as it is the only Einthoven lead along the heart horizontal axis. For normal zebrafish, we have no clear preference for either Einthoven lead II or III to portray the heart longitudinal axis. However, we ended up choosing lead II over lead III for two reasons. First, in normal healthy humans, the R wave in lead II is invariably prominent. As mentioned above, the mean normal human main axis aligns perfectly with lead II axis at +60° (Proger and Davis, 1930; Hiss et al., 1960; Simonson, 1972). In contrast, respiratory variation in QRS morphology and amplitude is most pronounced in lead III, hence lead III is less desirable (Proger and Davis, 1930; Einthoven et al., 1950; Surawicz and Knilans, 2008). Second, for zebrafish, a wealth of normal ECG data already exists for zebrafish standard lead rII, which documents that, like the normal human R wave in lead II, the normal zebrafish R wave in lead rII is also invariably prominent (Figure 3C, *left panel*). However, apart from the stability of the ECG wave size and polarity in a given lead, known or suspected cardiac pathology may dictate the choice of which two Einthoven leads to record and measure. For example, zebrafish lead III may be preferable to lead II in characterizing right axis deviation in a zebrafish model, say of right trabecular-bundle block.

### Relation of Electrical Axis to Anatomical Axis and Pump Physiology

A key determinant of the translational significance of the adult zebrafish electrical axis is how it relates to anatomical axis and pump physiology and how this structure-function relationship compares to that in humans. Yet, what is the value of the human electrical axis as an index of alterations in human anatomical axis in the frontal plane? The extensive body of literature on the relation between human electrical axis and anatomical axis, particularly in cases of electrical axis deviation, is controversial and forebodes another source of confusion in studies of zebrafish electrical axes, particularly in zebrafish disease models at risk for QRS axis deviation. Briefly, the relation between human electrical axis and anatomical axis is complex, non-linear, and depends on several factors (Dieuaide, 1921; Proger and Davis, 1930; Guntheroth et al., 1961). Minor factors, such as alterations in human body position or heart position (except situs inversus), have little effect on human electrical axis (Dieuaide, 1921; Proger and Davis, 1930). Variations in arrangements of human cardiac conduction system or disturbances of atrioventricular conduction have variable effects on electrical axis of diseased hearts of normal size (Proger and Davis, 1930). Overall, despite imperfect correlations, current consensus is that marked ventricular hypertrophy or enlargement is the most predominant factor influencing electrical axis deviation (Einthoven, 1906; Bridgman, 1915; White and Burwell, 1924; Proger and Davis, 1930; Scott, 1960).

Despite apparent simplicity, the zebrafish heart shares many common anatomical features with the mammalian heart (Hu et al., 2000). In adult zebrafish, the sinoatrial node, containing specialized pacemaker cells, is located at the junction of the sinus venosus with the posterior portion of the atrium (Sedmera et al., 2003; Tessadori et al., 2012). The atrium encircles the dorsal side of the ventricle, which is positioned anteroventrally in the thoracic cavity (Hu et al., 2001). The atrioventricular valve, connecting the atrium to the ventricle, is positioned anteroventrally to the atrium and dorsomedially to the ventricular base (Hu et al., 2001; Sedmera et al., 2003). From the atrioventricular ring, two main trabecular bundles connect to the ventricular apex (Sedmera et al., 2003). In this study, we found that the mean normal P frontal axis was −70°, in agreement with an atrial anatomical axis directed from the sinoatrial node posteriorly to the atrioventricular valve anteriorly. As mentioned above, we found that the mean normal QRS frontal axis was −70°, in agreement with a ventricular anatomical axis also directed from the apex posteriorly to the base anteriorly. Thus, in normal adult zebrafish, the concordance between the P and QRS frontal axes correlates at least in part with the posteroanterior concordance between the anatomical frontal axes of its atrium and ventricle.

However, interspecies differences in anatomical orientation, hence in anatomical nomenclature, may present common sources for confusion. Mindfulness of interspecies anatomical differences is warranted to prevent potential ECG misinterpretations of electrical axes. For example, “anterior” and “posterior” refer to human “ventral” and “dorsal,” respectively, but to zebrafish “cranial” and “caudal,” respectively. Thus, the anteroposterior axis of the zebrafish heart can be captured by limb leads (I-III, aV_*R*_, aV_*L*_, and aV_*F*_) in the frontal plane, but not by precordial leads (V_1_–V_6_) in the transverse plane. Additionally, in humans, because the left atrium, mitral valve, left ventricle, and aortic valve all connect along the heart longitudinal axis in a cranial-to-caudal direction, the heart chambers thus share the same cranial-caudal anatomical axis along which blood is pumped whereas human P and QRS axes also align in series craniocaudally. In contrast, in adult zebrafish, only the ventricle, bulboventricular valve, and bulbus arteriosus connect along the heart longitudinal axis in a caudal-to-cranial direction, but the atrium, atrioventricular valve, and ventricle connect along the heart transverse axis in a dorsal-to-ventral direction. The first ramification of these dual anatomical axes is the corresponding dual directions for blood pumping. The second ramification is that although the P and QRS axes also align in adult zebrafish along the heart longitudinal axis as they do in humans, zebrafish electrical axes align in the reverse, caudocranial direction and in parallel (rather than in series).

### Tissue Mechanisms: Electrical Propagation in the Frontal Plane

#### Intraspecies Comparison

In this study, one key similarity between the two Einthoven’s triangles is the intra-lead polarity concordance of all three ECG components, indicating that the three electrical heart axes align. The alignment of zebrafish P-wave and QRS axes indicates that, like humans, zebrafish atrial and ventricular activation proceed in the same direction. The alignment of zebrafish QRS and T-wave axes indicate, that like humans, zebrafish ventricular activation and ventricular repolarization proceed in opposite directions. These findings are consistent with our prior findings using *in vivo* bipolar single-lead ECG recording and ex vivo voltage-sensitive fluorescent epicardial and transmural optical mapping (Zhao et al., 2020).

#### Interspecies Comparison

Another key similarity between zebrafish and humans is the interspecies positive concordance in lead I for all three ECG components. This finding supports the translational relevance of lead I, an uncommon zebrafish lead, in reflecting the differences in electrical activity between the right and left sides of the ventricular base (or of the heart frontal short axis). In contrast, one key difference between the two Einthoven’s triangles is the interspecies discordance in lead II: predominantly negative for zebrafish vs. positive for humans. This explains why, in the conventional practice of *in vivo* surface ECG recording for adult zebrafish, lead rII is the most popular (and potentially confounding) choice as the bipolar single-lead standard to reverse the polarities of the three zebrafish ECG waveforms and simulate concordance with human lead II. Therefore, a direct ramification of the interspecies lead-I positive concordance and lead-II discordance is that the three electrical axes of zebrafish and human hearts do not align in the same quadrant but are instead reflections across the horizontal (or 0° in the Cabrera system). In other words, zebrafish and human atrial depolarization, ventricular depolarization, and ventricular repolarization normally propagate in mirror images across the horizontal (Figure 3D).

### Zebrafish Longer Ventricular Action Potential Plateau

Importantly, this study unveils that QTc duration is the only ECG component measuring the same in both species whereas QRS and T-wave durations are 30–35% shorter in zebrafish than in humans. Direct STc-duration measurements confirmed our hypothesis that as compensation, STc segment is 30% longer in zebrafish than in humans. Thus, compared to humans, zebrafish ventricular activation and late repolarization are faster, but early repolarization is slower. In other words, compared to humans, zebrafish ventricular action potential upstroke and phase 3 are shorter, but zebrafish ventricular plateau, which reflects ventricular repolarization, is longer. This critical tissue and cellular mechanism insight may portend clinical implications for the adult zebrafish heart pharmacological responses as a drug-screening platform for QT prolongation and as an electrophysiological model for human arrhythmias studies.

What is the likely molecular basis for the longer zebrafish ventricular action potential plateau? While identifying the exact responsible ionic-current mechanism is beyond the scope of this study, the rich body of evidence in existing literature allows educated predictions. Normal ventricular repolarization relies on a critical balance and complex interaction during the action potential plateau between repolarizing outward currents (such as multiple types of K^+^ currents) and depolarizing inward currents (such Ca^2+^ currents) (Nerbonne and Kass, 2005; Nguyen et al., 2015). For example, compared to larger mammals (including humans) and to zebrafish, mouse ventricular myocytes have higher transient outward K^+^ current (I_to_) density, which accounts for their curtailed action potential plateau (Bondarenko et al., 2004; Nguyen et al., 2015). Hence, compared to the resultant triangular-shaped mouse ventricular action potential, the rectangular-shaped zebrafish ventricular action potential has higher clinical relevance as it more closely resembles the rectangular-shaped human ventricular action potential with both zebrafish and human ventricular tissue having significantly longer plateau phase than mouse ventricular tissue (Verkerk and Remme, 2012; Vornanen and Hassinen, 2016). However, here we found that the ventricular plateau phase is longer in zebrafish than in humans. This finding highlights at least three known interspecies differences in the ionic current determinants of the ventricular action potential morphology and duration. Different from normal adult human ventricular myocytes, normal adult zebrafish ventricular myocytes express no I_to_, negligible slowly activating component of the delayed rectifier K^+^ current (I_Ks_), but robust T-type Ca^2+^ current (Nemtsas et al., 2010; Verkerk and Remme, 2012; Wu et al., 2014; Vornanen and Hassinen, 2016; Echeazarra et al., 2021). Any of these three differences, in isolation or combination, can account for zebrafish longer lasting plateau phase of the ventricular action potential.

### Zebrafish Larger Relative Amplitude of the P Wave

The difference of zebrafish and human heart sizes by two orders of magnitude explain the differences in their voltage amplitudes. In this study, the human-to-zebrafish ratios of mean amplitudes from all Einthoven leads for the P wave, QRS complex, and T wave were 3, 7, and 58, respectively. This finding highlight the significant difference between zebrafish and human in inter-chamber comparative sizes. While adult zebrafish atrium is roughly the same size as adult zebrafish ventricle (Singleman and Holtzman, 2012), adult human atria are only one-third of adult human ventricular sizes. This anatomical difference explains why in this zebrafish age group (12–18 months of age), the normal zebrafish P wave was the second largest of the three ECG components whereas normal human P wave is consistently the smallest of the three ECG components. The relative large amplitude of zebrafish P wave may present an advantage of the adult zebrafish atrium as a model for human atrial electrophysiology and arrhythmias.

### Study Limitations

This study faced technical limitations. First, zebrafish inability to cooperate during *in vivo* ECG recording necessitated the need for anesthesia with known cardiorespiratory adverse effects. Second, zebrafish small myocardial mass required keeping Einthoven’s triangle tight around the heart to maximize signal-to-noise ratio, thus compounding the challenge of quadruple-electrode placement into a small zebrafish chest. This explains why this study was the first to attempt *in vivo* dual-lead surface ECG in zebrafish. While we could extend the coverage flexibility of our current instrumental setup (29-gauge electrodes; ADInstruments) to record *in vivo* surface ECG from larger adult mammals (rabbits and rodents) to tiny adult zebrafish, the physical limitation of zebrafish chest size necessitated limiting zebrafish age to ≥4 months for single-lead ECG and ≥6 months for dual-lead ECG. Future engineering improvements to lighten electrode wirings and to prevent slippage prevention of smaller electrodes (e.g., 32-gauge) will allow extension of these age ranges to younger zebrafish.

Lastly, our study was unlikely powered to detect all ECG sex differences in humans or zebrafish. Additionally, sex determination is more complex in zebrafish than humans (Liew and Orbán, 2014). Unlike human sex determination, zebrafish sex is polygenic. Zebrafish sex is not determined based on sex chromosomes alone but results from allelic combinations of several loci dispersed throughout the genome. Thus, several autosomal genes may determine zebrafish sex with or without contribution from sex chromosomes. In humans, sex differences impact the prognostic significance of ECG presentations, for example following myocardial infarction (Mieszczanska et al., 2008). Given the complexity of sex determination in zebrafish, the value of zebrafish as a model for sex differences in post-injury cardiac prognosis remains to be determined.

## Data Availability Statement

The original contributions presented in the study are included in the article/supplementary material, further inquiries can be directed to the corresponding author.

## Ethics Statement

The animal study was reviewed and approved by the UCLA Institutional Animal Care and Use Committee.

## Author Contributions

YZ, CC, MY, TI, and AL performed the experiments, analyzed the data, and prepared the figures. TN conceived the study, designed the experiments, and prepared the manuscript. All authors approved the manuscript final version and agreed to be accountable for all aspects of the work.

## Conflict of Interest

The authors declare that the research was conducted in the absence of any commercial or financial relationships that could be construed as a potential conflict of interest.

## Publisher’s Note

All claims expressed in this article are solely those of the authors and do not necessarily represent those of their affiliated organizations, or those of the publisher, the editors and the reviewers. Any product that may be evaluated in this article, or claim that may be made by its manufacturer, is not guaranteed or endorsed by the publisher.
